# Purification and characterization of lipoxygenase from mung bean (*Vigna radiata* L.) germinating seedlings

**DOI:** 10.1007/s13205-016-0427-5

**Published:** 2016-05-17

**Authors:** Raveendra Aanangi, Kasi Viswanath Kotapati, Bhagath Kumar Palaka, Thyagaraju Kedam, Nirmala Devi Kanika, Dinakara Rao Ampasala

**Affiliations:** 1 Department of Biochemistry, Sri Venkateswara University, Tirupati, 517 502 Andhra Pradesh India; 2 Centre for Bioinformatics, School of Life sciences, Pondicherry University, Puducherry, 605014 India

**Keywords:** Chromatofocusing, Circular dichroism, Mung bean, Plant lipoxygenases, Protein purification

## Abstract

This study reports purification and characterization of lipoxygenase protein from mung bean germinating seedlings. Lipoxygenases (LOXs) are key enzymes in seed germination. The purified mung bean LOX has resolved into two peaks by chromatofocusing, one has highest LOX activity with an isoelectric point of 5.84 and the other has lowest LOX activity with an isoelectric point of 5.52. The purified LOX has molecular mass of approximately 97 kDa and showed high activity with linoleic acid than linolenic acid and arachidonic acid. The optimal activity of LOX was observed at pH 6.5 and temperature 35 °C. Far-UV circular dichroism (CD) studies revealed that the purified mung bean LOX possess secondary structural elements with significant α-helix and β-strands. Further, the secondary structure of mung bean LOX was stable up to 60 °C at pH 6.5. Biophysical and chemical properties of the mung bean LOX are similar to the other legume LOXs and may be considered as type-1 LOX.

## Introduction

Lipoxygenases (LOXs, linoleate: oxygen oxidoreductases, EC: 1.13.12.11) represent a large gene family of non-heme, non-sulfur iron or manganese containing dioxygenases which are ubiquitously distributed throughout nature indicating the biological and evolutionary importance of these enzymes (Joo and Oh 2012). LOXs catalyze the regio- and stereo-specific dioxygenation of polyunsaturated fatty acids (PUFAs) containing (*1Z*, *4Z*)-pentadiene system (Kuhn and Thiele 1999). Evidences suggested that the primary products generated by these enzymes are called as hydroperoxides, which act as substrates for the synthesis of short chain carbonyl compounds, involved in plant growth, host–pathogen interactions, defense, development and senescence (Brash 1999; Gfeller et al. 2010; Siedow 1991). LOXs are also known to play a major role in production of volatile substances, which influence the flavor and aroma of food from the plants and have wide applications in food industry as they play important role in maintaining food quality and the aroma of food grains (Suzuki et al. 2010).

Germination is the most critical step in the life cycle of spermatophytes and represents the entry of plants into the ecosystem, during which a seed from a dormant embryonic state enters into a highly dynamic phase leading to seedling establishment (Weitbrecht et al. 2011). Germination begins with imbibition and terminates with seed coat rupture and radicle protrusion through the endosperm (Kong et al. 2015). During germination, lipid bodies are degraded in seeds by a new set of proteins, among which, LOXs are playing an important role (Feussner et al. 2001).

During the past few decades, many isoforms of LOX were identified from different plants and their physical and enzymatic properties are characterized. Although occurrence of LOXs in vegetables and fruits is known, it has been reported that legumes are rich source for LOXs, among them soybean seed LOXs were well studied at molecular level (Feussner and Wasternack 2002; Kolomiets et al. 2001). Three isoforms of LOX were identified and characterized from soybean seeds based on their pH optimum, substrate specificity, product formation, kinetic parameters and enzyme stability (Axelrod et al. 1981; Clemente et al. 2000; Kolomiets et al. 2001). Apart from soybean, other legumes have also been reported as good source of LOX proteins. Legumes are inexpensive source of LOX proteins and particularly high level of LOX activities were identified from various legume seeds (Rao et al. 1998). Mung bean was identified as a novel source of LOX in the natural production of green-note aroma compound, hexanal, it is also considered as a cheap and readily available staple food in Asia (Chow et al. 2007). Further, mung bean and its seedlings have been used as a source of hydroperoxide lyase enzyme (Rehbock et al. 1998). Previously, high levels of LOX activity have been reported in germinating seedlings of mung bean (Devi et al. 2005; Rao et al. 1998). Earlier we reported a full-length LOX from mung bean germinating seedlings, during developmental stage more level of LOX expression was observed (Kotapati et al. 2015). However, no reports were available to date on purification and characterization of LOX from mung bean.

In the present study, considering the importance of LOX proteins in food industry, a report on the isolation and biochemical properties of purified LOX from mung bean seedlings is presented.

## Materials and methods

### Plant material and chemicals

The mung bean seeds were obtained from Sri Venkateswara Agricultural University, Tirupati. Soybean LOX, ETYA, Linoleic acid, Linolenic acid, Arachidonic acid, Sephadex G-100, DE-52, Poly Buffer Exchangers 94 (PBE-94), Phenyl methyl sulfonyl fluoride (PMSF), EDTA, Acrylamide, Bis-acrylamide, Coomassie brilliant blue, Lauryl sulphate (SDS) and protein size markers were procured from Sigma Chemicals Co (St. Louis, MO, USA). NDGA was a generous gift from department of chemistry, S.V. University. All other chemicals were reagent grade procured from Merck, Mumbai, India.

### LOX protein extraction and purification

Extraction and purification methods for mung bean LOX was adopted from Clemente et al. (2000) and Roopashree et al. (2006) with minor modifications. In brief, finely ground powder of mung bean seedlings was extracted with ice cold hexane to make it defatted. About 30 g of defatted fine powder was extracted with 300 ml of 50 mM sodium phosphate buffer, pH 6.8, at 4 °C for 3–4 h and centrifuged at 10,000×*g* for 30 min. The fine supernatant was dialyzed against 25 mM sodium phosphate buffer (pH 6.8) for 24 h with three buffer changes and centrifuged at 25,000×*g* for 20 min. The supernatant was dialyzed against 40 % poly ethylene glycol 20,000 for 16 h and then centrifuged at 25,000×*g* for 20 min. The dialyzed sample was applied to Sephadex G-150, gel filtration column (100 × 2.5 cm) and fractions were collected with a fraction size of 2.5 ml per tube at a flow rate of 20 ml/h. The active fractions were pooled and further purified by ion exchange chromatography (DEAE 52, column 3 × 30 cm). Bound protein was eluted using a linear salt gradient [0 mM (150 ml) to 300 mM (150 ml)] sodium phosphate buffer [pH 6.8] and fractions were assayed for protein and LOX activity. At the end of this purification step, two protein fractions were obtained, one with high and the other without LOX activities. To determine the isoelectric points of mung bean seedling LOX, the peak with high LOX activity fractions was pooled, concentrated and dialyzed to remove salt, centrifuged at 25,000*×g* for 20 min at 4 °C and the supernatant was applied on the PBE-94 chromatofocusing column (3 × 12 cm) which was saturated with 5 ml of gradient buffer (Poly buffer 94, 1: 8, pH 4.0) to create a pH gradient in column. The flow rate was adjusted to 8 ml/h and elute was collected in 1 ml per fraction. The protein in each fraction was read at 280 nm and assayed for LOX activity. The pH of each fraction was determined by using a KL-009 (1B) pocket size pH meter. All purification steps were performed at 4 °C until otherwise mentioned.

### SDS-PAGE

SDS-PAGE was performed according to the method of Laemmli (1970) using 12 % gels. The proteins were stained with Coomassie brilliant blue R-250 in methanol:water:acetic acid (60:30:10) for few hours and then washed in destaining buffer until protein bands appear.

### Activity staining

Sample containing 50–100 µg LOX protein of germinated seedlings extract was separated on 8 % polyacrylamide gel electrophoresis without adding SDS to the gel and running buffer (0.025 M Tris–HCl and 0.192 M glycine, pH 8.8) at 4 °C as suggested by Heydeck and Schewe (1984). In brief, following the isozymes separation, gels were washed briefly with phosphate buffer (pH 6.8), and incubated in substrate solution for 5 min at room temperature. After incubation the gels were washed quickly with 100 mM phosphate buffer (pH 6.8), and incubated in staining solution with *O*-diansidine-HCl (50 mg *O*-Diansidine-HCl dissolved in 10 ml absolute ethanol under heating and add this solution to 90 ml of 100 mM phosphate buffer, pH 6.8). The electropherogram which has LOX enzymes converts the fatty acid substrate into fatty acid hydroperoxides. These hydroperoxides oxidize both the amino groups of 3-3′ methoxybenzidine hydrochloride forming the chromophores. The chromophores give a reddish brown colour by which the presence of isoenzymes could be localized.

### Enzyme assay

Lipoxygenase activity was measured as previously described using Jasco V-530 UV–VIS spectrophotometer at 25 °C by monitoring the increase in absorbance over a period of time at 234 nm (Reddanna et al. 1990). The typical reaction mixture contains 2.8 ml of 50 mM sodium phosphate buffer (pH 6.5), appropriate volume of enzyme (10–100 µl) and the reaction was initiated by addition of substrate to the reaction mixture and maintained to have 250 µM for linoleic acid in the total volume. One unit of enzyme activity is defined as the µmol of hydroperoxide formed per min^−1^ mg^−1^ protein.

## Kinetic parameters

### Effect of pH and temperature

The optimum pH for mung bean LOX activity was investigated using linoleic acid as substrate at 25 °C within the pH range of 3.0–10.0. The effect of ionic strength on mung bean LOX enzyme activity was tested using different concentrations of sodium phosphate buffer (pH 6.5) from 10 to 200 mM. To figure out the optimum temperature of mung bean LOX enzymes, activity was measured using pre-incubated standard assay mixture for 10 min at the indicated temperature (20–80 °C). After incubation, samples were cooled to 25 °C and the remaining LOX activity was determined.

The kinetic parameters of mung bean seedling LOX were measured under standard assay conditions using substrate concentrations in the range of 2–400 μM. All determinations were done in duplicate and respective kinetic parameters were calculated from Line-weaver Burk (LB) plot.

### Effect of inhibitors on mung bean seedling LOX activity

Effect of two known LOX inhibitors on mung bean LOX activity was investigated under standard conditions using different concentrations of ETYA (0–300 µM) and NDGA (0–500 µM). The stock solutions of both inhibitors were prepared in 100 % alcohol. The inhibitors were pre-incubated with enzyme for 5 min and assay was performed as mentioned in previous sections.

### Circular dichroism (CD) studies of mung bean seedling LOX

Circular dichroism (CD) experiments were carried out using Jasco J-715 Polari meter. CD measurements were performed using homogeneous samples at a protein concentration of 60–80 μg/ml in 10 mM phosphate buffer at appropriate pH as described by (Barone et al. 1999). Far-UV CD spectra were recorded from 200 to 250 nm using 0.1 cm light path quartz cuvettes under continuous nitrogen flow. A special acquisition spacing of 0.1 nm was used and each spectrum was averaged four times and smooth curves were made. The thermal stability of mung bean LOX was investigated from 25 to 80 °C at different pH conditions.

## Results and discussion

### Purification and characterization

**Fig. 1:**
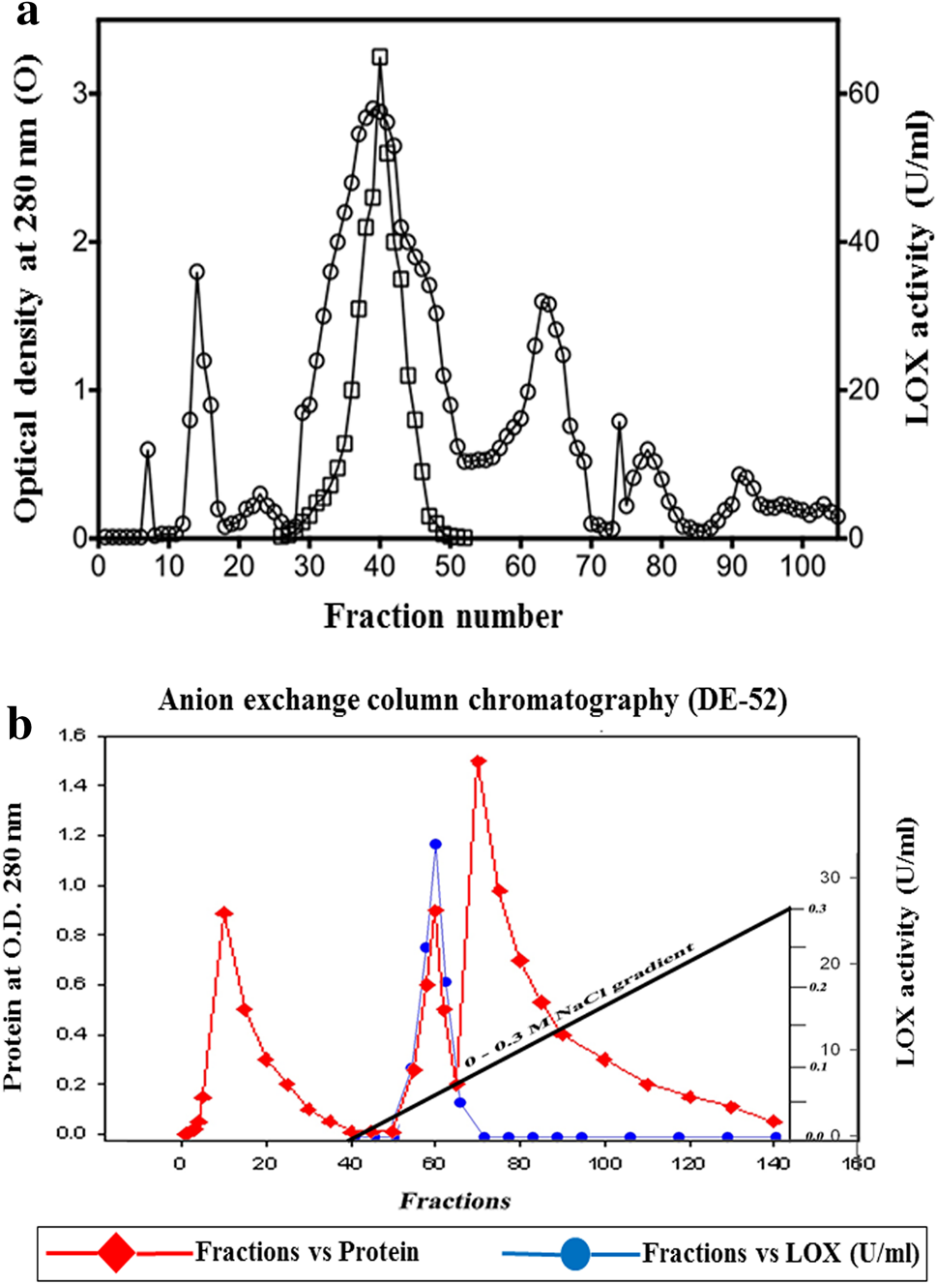
**a** Gel filtration chromatography of mung bean LOX on Sephadex G-150 column. Fractions of 5 ml were collected and assayed for protein at 280 nm and LOX activity at 234 nm. **b** Anion exchange chromatography of mung bean LOX on DE-52 column, 0.0–0.3 M NaCl salt gradient was used to elute LOX protein from the column. Fractions of 2.5 ml were collected and assayed for protein at 280 nm and LOX activity at 234 nm

**Fig. 2:**
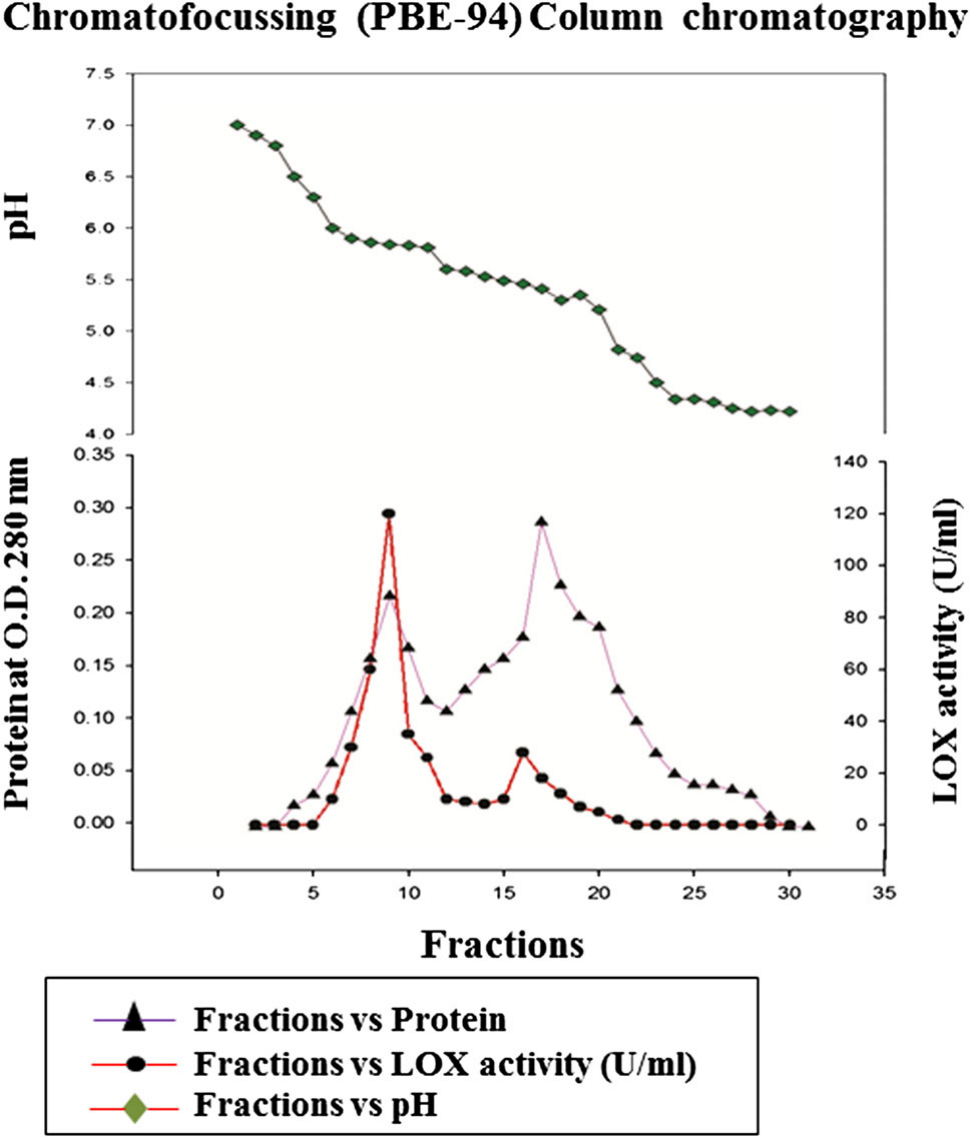
Isoelectrofocussing column chromatography of mung bean LOX on PBE-94 column. Fractions of 1.0 ml were collected and assayed for protein at 280 nm and LOX activity at 234 nm

**Fig. 3:**
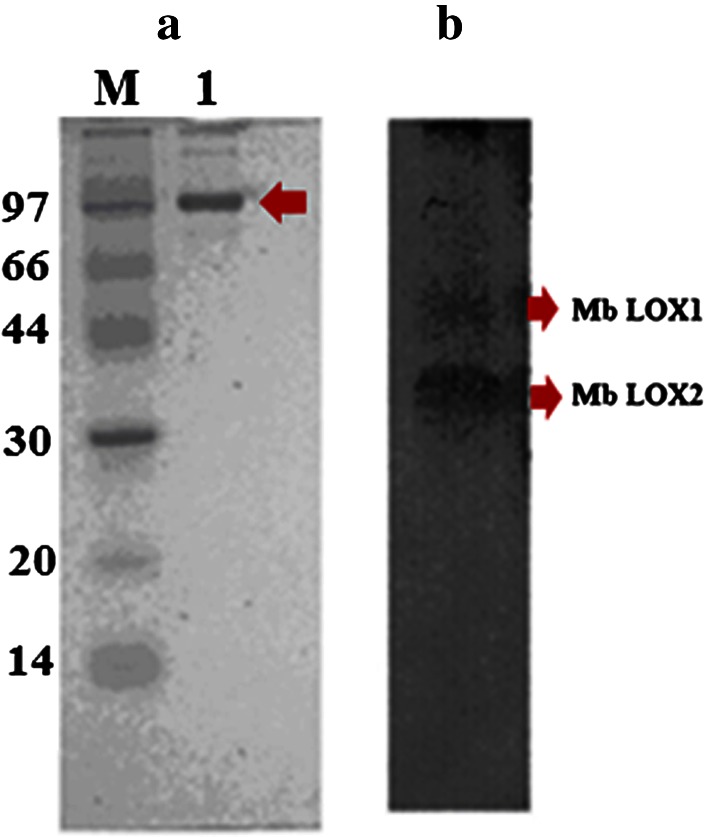
**a** SDS-PAGE analysis of LOX purified from mung bean seedlings. Lane *M* molecular weight standards (in kDa). Lane *1* Anion exchange (DE-52) Purified mung bean LOX (peak1). **b** Native PAGE analysis- Mung bean LOX isoenzymes (Mb LOX1 and Mb LOX2) stained with *O*-Diansidine-HCl under native conditions run at 4 °C

The enzyme was purified to 27folds by chromatographic (gel filtration, ion exchange and chromatofocusing) methods. The detailed description of mung bean LOX purification is given in Table 1. Loss in LOX activities (40–50 %) of broad bean, french bean and horse bean was reported when ammonium sulfate fractionation was used as an initial purification step (Feussner and Wasternack 2002). Hence ammonium sulfate precipitation was avoided in the initial step of purification. Instead, an albumin-enriched fraction was obtained as mentioned by Kuo et al. (2006) and is considered as initial step for further purification. Proteins with purification fold 3.5 were obtained in albumin fraction. During gel filtration chromatography a broad single peak of mung bean LOX protein was eluted with purification fold of 3.8 and a specific activity of 22.24 U/mg protein. Finally, anion exchange chromatography yielded two protein fractions upon NaCl salt gradient elution, one with high and the other without LOX activity with purification fold of 4.6 with 26.7 U/mg specific activity (Fig. 1a, b; Table 1) and which was considered for further characterization. The mung bean LOX active protein fraction was resolved into two isozymes on chromatofocusing (Fig. 2). The first isozyme has an isoelectric point of 5.84 and showed high specific activity (160 U/mg) with purification fold of 27. The second has an isoelectric point of 5.52, showed low specific activity (11.11 U/mg) and with purification fold of 2.0, which eventually lost its activity during the purification process. The p*I* values of mung bean LOX are closely related to English pea and soybean LOX isoenzymes (Eriksson and Svensson 1970). The SDS-PAGE purified mung bean LOX (first isozyme fraction) showed a single band with an approximate molecular mass of 97 ± 5 kDa and greater than 90 % purity (Fig. 3a). The molecular mass of mung bean LOX is similar to broad bean, faba beans, soybean, durum wheat and pea LOXs as reported (Barone et al. 1999; Clemente et al. 2000). Activity staining on native PAGE of mung bean seedlings extract showed two brown enzymatically active bands which indicate the presence of two LOX isoenzymes during seedling growth (Fig. 3b). Based on the activity staining, presence of multiple bands suggest that perhaps two or more isoenzymes will be expressed in later stages of plant development and each will play important roles in plant growth and defense (Haydar and Hadziyev 1973).

**Table 1:** Summary of purification methods employed for lipoxygenase purification from mung bean germinating seedlings

Method	Protein (mg)	Total activity (units)	Specific activity (U/mg)	Yield (%)	Purification (fold)
Crude extract	5183	30,224	5.83	100	1
Albumin fraction	1040	20,976	20.20	69.0	3.5
Sephadex G-100	625	11,900	19.04	39.4	3.3
DEAE-DE-52	390	10,400	26.67	34.4	4.6
Chromatofocussing (PBE-94)					
Peak-1 (pI-5.84)	1.25	200	160.0	1.0	27.4
Peak-2 (pI-5.52)	0.63	7.0	11.11	0.02	2.0

### Kinetic studies

**Fig. 4:**
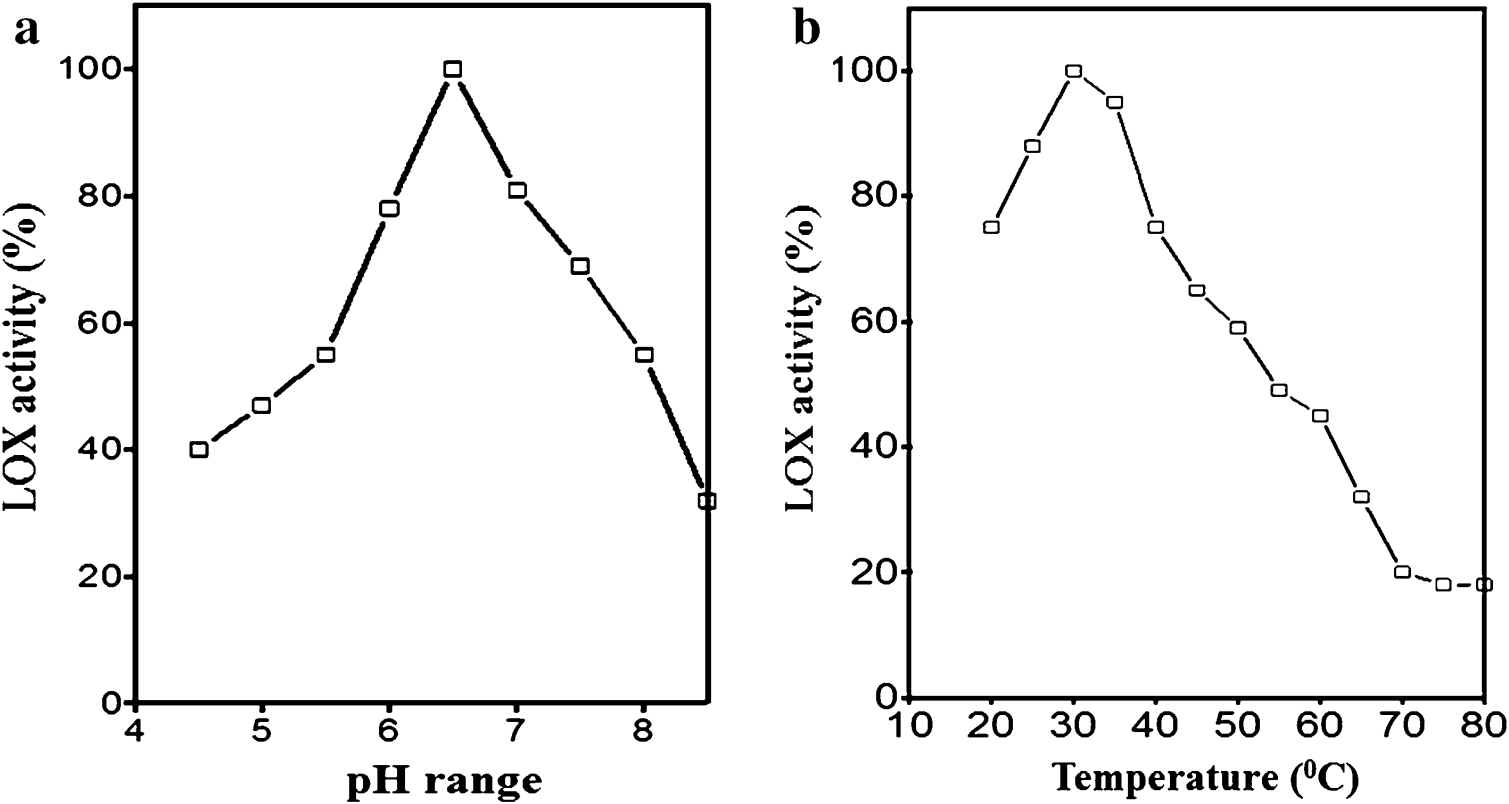
Optimal conditions for mung bean LOX activity. **a** Effect of pH on purified mung bean LOX. Different buffers were prepared at 50 mM concentration and adjusted to defined pH values. **b** Effect of temperature on purified mung bean LOX. Samples were pretreated for 10 min at given temperature and then assayed for LOX activity under standard conditions

The optimal pH for mung bean LOX was found to be 6.5. More than 50 % loss in mung bean LOX activity was observed below pH 4.5 or above pH 8.0; however, considerable LOX activity was noted with in a pH range 4.5–8.0 (Fig. 4a). The effect of ionic strength on mung bean LOX enzymatic activity revealed the maximal activity at 50 mM concentration of sodium chloride (Data not shown). Although appreciable LOX activities were observed between 50 and 150 mM salt concentrations, reduced enzyme activities were noted below 50 mM salt concentration (Fig. 4a). Mung bean LOX showed maximal activity at 35 °C and retain its activity up to 60 °C (Fig. 4b). However, significant decrease in enzyme activity was observed at higher temperature (over 50 °C). The optimal pH and temperature studies of mung bean LOX are closely identical to other plant LOXs (Feussner and Wasternack 2002; Kolomiets et al. 2001).

**Fig. 5:**
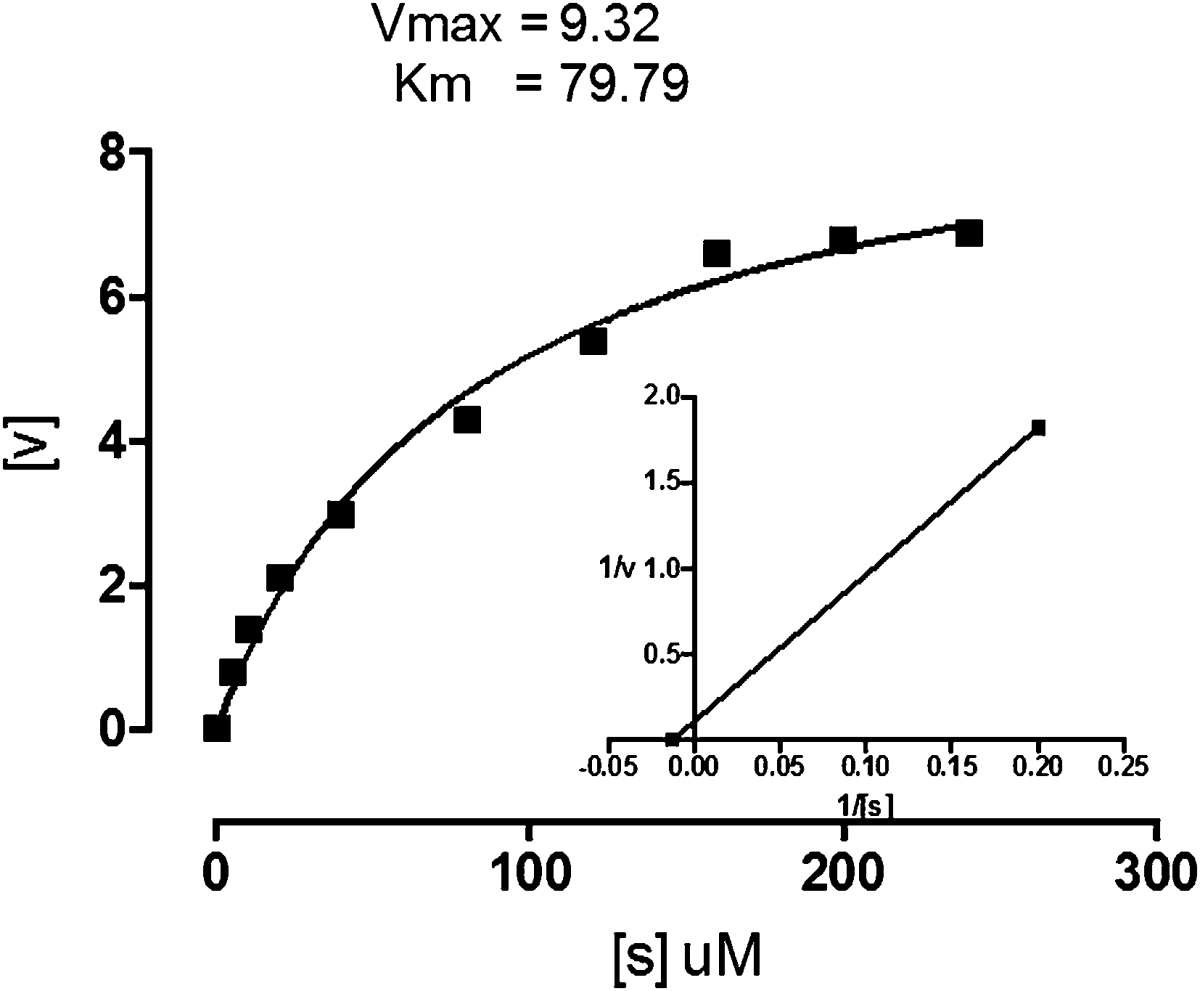
Effect of substrate concentration on mung bean LOX and determination of Vmax and Km. Data showed for linoleic acid as substrate for mung bean LOX

Substrate specificity of mung bean LOX was determined using linoleic, linolenic and arachidonic acids, and kinetic constants were obtained (Table 2). The apparent *K*_m_ of mung bean LOX for three substrates tested is 79.79, 135.5 and 253.1 μM (Fig. 5). Given data suggest that linoleic acid is preferred substrate for mung bean LOX when compared with linolenic and arachidonic acids. The apparent *K*_m_ and *V*_max_ values of mung bean LOX are closely relevant with the other legumes like germinating barley (van Aarle et al. 1991), soybean (Axelrod et al. 1981; Dainese et al. 2010), durum wheat semolina (Barone et al. 1999) and broad bean (Clemente et al. 2000) LOX isoenzymes.

**Table 2:** Characteristics of lipoxygenase purified from mung bean seedlings

Parameter	Mung bean LOX
pH optimum	6.5
Isoelectric points (pI)	5.84 and 5.52
Temperature (°C)	35–40
Ionic strength (mM)	50
*K*_m_ (µM)	
Linoleic acid	79.79
Linolenic acid	135.5
Arachidonic acid	253.1
*V*_max_ (µmol/min)	
Linoleic acid	9.32
Linolenic acid	7.31
Arachidonic acid	8.63
IC50 (µM)	
ETYA	20
NDGA	48
Structure	Alpha-helix and beta sheet
Products (absorption max)	234 and 278
Inflection point	60 °C (pH 6.8)
	50 °C (pH 9.0 and 5.0)

**Fig. 6:**
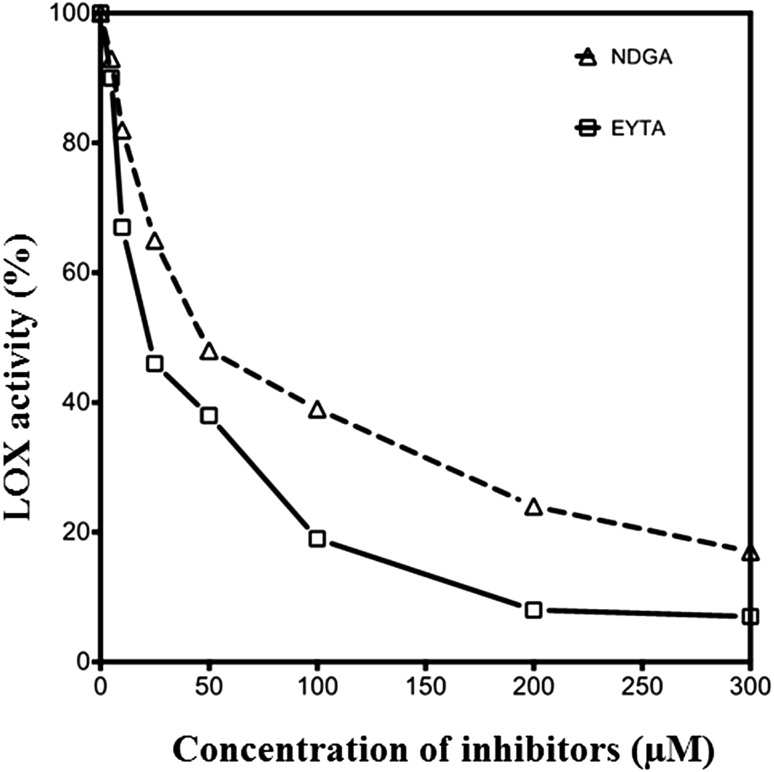
Effect of inhibitors on mung bean LOX activity. *Square* ETYA and *upright triangle* NDGA

Two well-known LOX inhibitors, ETYA and NDGA, were tested to investigate the effect on mung bean LOX (Fig. 6). The calculated IC_50_ of ETYA and NDGA with mung bean LOX were 18.18 ± 1.2 and 48.07 ± 3.2 µM, respectively. The inhibition kinetics of two LOX inhibitors, ETYA and NDGA, followed competitive and non-competitive mode of inhibition, respectively. Similar kind of inhibition constants were reported for soybean type-1 LOX and type-2 LOX, and human LOX-5 (Kohyama et al. 1997; Kolomiets et al. 2001; Nagabhushana et al. 2002).

### Circular dichroism (CD) studies

**Fig. 7:**
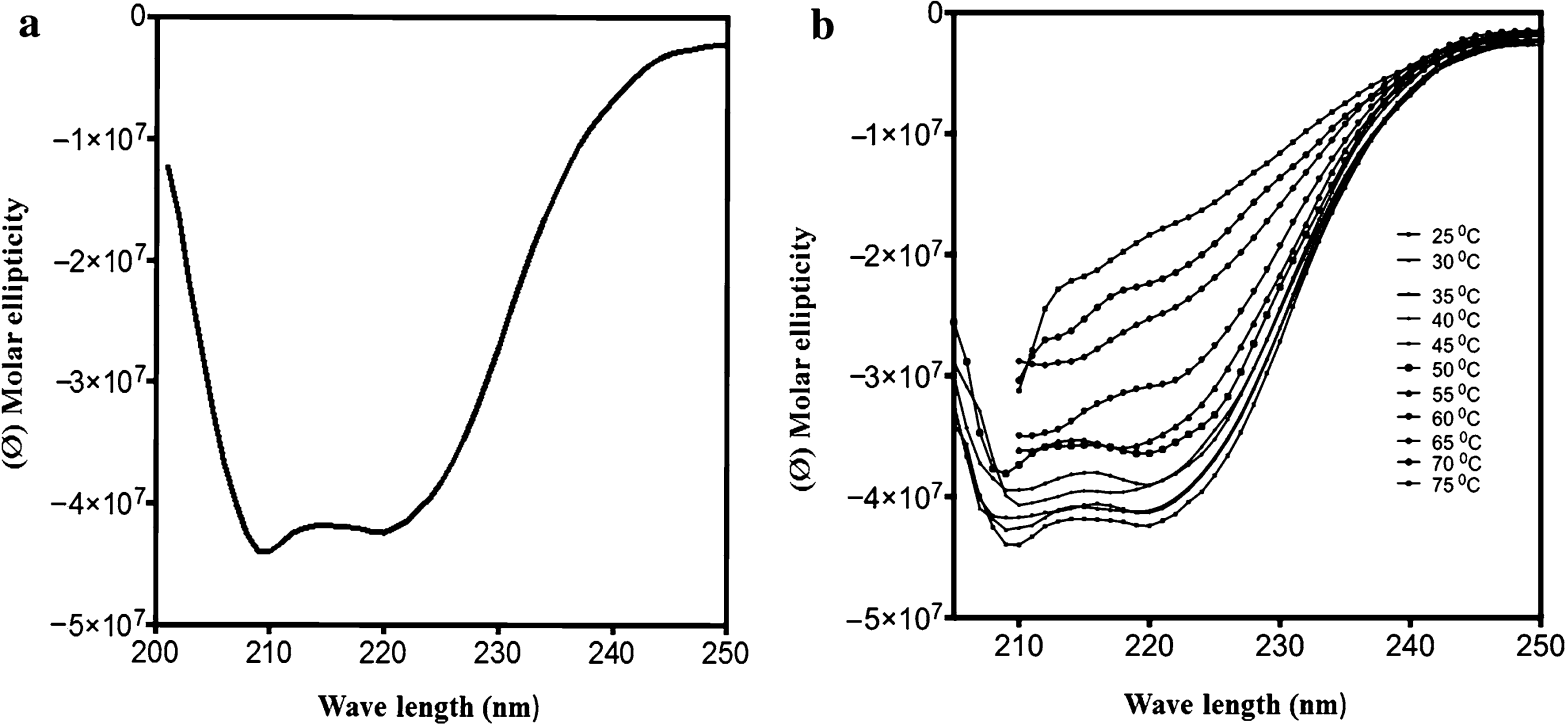
Circular dichroism analysis of purified mung bean LOX. **a** Far-UV CD spectra. Measurements were made at 25 °C in 10 mM phosphate buffer (pH 6.5). **b** Temperature effect on mung bean LOX as function of secondary structure. Mung bean LOX was pre-incubated at given temperature for 10 min and measurements were made in 10 mM phosphate buffer (pH 6.5)

Far UV-circular dichroism spectra of mung bean LOX showed a negative dip at 208 and 222 nm, indicating the existence of predominant secondary structure with significant α-helix and β-strands (Fig. 7a). Further, temperature effect on mung bean LOX as function of its secondary structure at optimal pH showed that the secondary structures were stable up to 60 °C and the secondary structures were destabilized upon further increase of temperature (Fig. 7b). Thermal stability of mung bean LOX was also depicted by measuring the dichroic activity at 222 nm as a function of temperature. A Sigmoid decrease in ellipticity at 222 nm was observed with increase in temperature and the inflection point of 60 °C at optimal pH. The CD data of mung bean LOX is consistent with LOX activities measured at different temperatures whereas loss in enzymatic activities was observed over 60 °C at optimal pH. These data suggest that, the mung bean LOX secondary structure is pH and temperature dependent and significant loss in enzymatic activity is due to loss in secondary structure of protein (Graff et al. 1990).

## Conclusions

In conclusion, we have purified and characterized the mung bean lipoxygenase by biophysical and biochemical analysis. Mung bean LOX that we purified has similar properties like soybean LOX1 type. Our findings also suggest that presence of multiple isoforms in seedlings perhaps play key roles in developmental stages. Although the predominant isoform in mung bean is LOX1, detailed characterization of other isoforms can also be considered useful as the by-products of LOX subtypes have industrial applications. Our recent cloning data also suggest the presence of multiple isoforms in mung bean. As mung bean is one of the food sources, the present study will serve as basis for detailed analysis of mung bean LOX isoforms and their roles in mung bean related food products.

## Abbreviations

LOX: Lipoxygenase
PUFAs: Polyunsaturated fatty acids
ETYA: Eicosatetraenoic acid
DE-52: Diethyl amino ethyl cellulose
NDGA: Nordihydroguaiaretic acid
CD: Circular dichroism
TBA: Thiobarbituric acid
TCA: Trichloro acetic acid
MDA: Malondialdehyde
X-Gal: 5-Bromo-4-chloro-3-indolyl β-d-galactopyranoside
PMSF: Phenylmethylsulfonyl fluoride
